# Association of the Meaningful Use Electronic Health Record Incentive Program With Health Information Technology Venture Capital Funding

**DOI:** 10.1001/jamanetworkopen.2020.1402

**Published:** 2020-03-24

**Authors:** Samuel Lite, William Joseph Gordon, Ariel Dora Stern

**Affiliations:** 1 Harvard Business School, Boston, Massachusetts; 2 Department of Medicine, Brigham and Women’s Hospital, Boston, Massachusetts; 3 Harvard Medical School, Boston, Massachusetts; 4 Partners HealthCare, Boston, Massachusetts; 5 Harvard-MIT Center for Regulatory Science, Boston, Massachusetts

## Abstract

**Importance:**

Although the Health Information Technology for Economic and Clinical Health (HITECH) Act has accelerated electronic health record (EHR) adoption since its passage, clinician satisfaction with EHRs remains low, and the association of HITECH with health care information technology (IT) entrepreneurship has remained largely unstudied.

**Objective:**

To determine whether the passage of the HITECH Act was associated with an increase in key measures of health care IT entrepreneurship.

**Design, Setting, and Participants:**

This economic evaluation of venture capital (VC) activity in the US from 2000 to 2019 examined funding trends in health care IT, EHR-related companies, and all VC investments before and after the passage of HITECH. A difference-in-differences analysis compared investments in health care IT companies with those of companies in 3 categories: general health care (non-IT), IT (non–health care), and all US VC transactions. Data were analyzed from September 2018 to August 2019.

**Exposures:**

Venture capital funding received by US companies before and after the HITECH Act.

**Main Outcomes and Measures:**

Venture capital investment in health care IT companies and the proportion of those investments going to seed-stage companies, a proxy for very early-stage entrepreneurship and innovation.

**Results:**

The data included 70 982 investments, of which 9425 (13.3%) were seed stage, 10 706 (15.1%) were early stage, and 50 851 (71.6%) were growth stage. After passage of the HITECH Act, investment in both health care IT companies and EHR-related companies increased at a rate much faster (13.0% and 11.4%, respectively) than VC as a whole (6.9%). In addition, the proportion of investments going to seed-stage health care IT companies increased compared with both overall VC investments and non-IT health care investments. Health care IT companies saw increased probabilities of transactions being seed-stage of 5.1% (SE, 2.2%; 95% CI, 0.8% to 9.3%; *P* = .02) compared with the entire sample of VC transactions and 13.6% (SE, 1.9%; 95% CI, 9.9% to 17.2%; *P* < .001) compared with non-IT health care VC transactions. Health care IT had essentially 0 increased probability of a transaction being seed stage compared with IT companies outside health care (−0.8% probability; SE, 2.4%; 95% CI, −5.4% to 3.9%; *P* = .75).

**Conclusions and Relevance:**

Although widespread clinician dissatisfaction with EHR systems remains a challenge, the HITECH Act’s incentive program may have catalyzed early-stage entrepreneurship in health care IT, suggesting an important role for incentives in promoting innovation.

## Key Points

**Question:**

What is the association between the Health Information Technology for Economic and Clinical Health Act, a federal subsidy program for electronic health record adoption, and venture capital investments and innovation in health care information technology?

**Findings:**

This economic evaluation used observational data on venture capital activity in the US and a difference-in-differences design and found that investments in health care information technology and electronic health record companies increased at a much faster rate than venture capital investments as a whole and that these investments were more likely to be seed-stage (very early) investments compared with all other industries and 13.6% more likely to be seed stage compared with non–information technology health care investments.

**Meaning:**

The Health Information Technology for Economic and Clinical Health Act’s incentive program was associated with increased investment in health care information technology and electronic health record–related companies compared with other industries, with an emphasis on early-stage companies, suggesting an important role for incentives in promoting innovation.

## Teaser

This economic evaluation study examines whether the passage of the Health Information Technology for Economic and Clinical Health Act was associated with an increase in key measures of health care information technology entrepreneurship.

## Introduction

In February 2009, as part of the American Reinvestment and Recovery Act—also known as the Obama administration’s economic stimulus package—the US Congress passed the Health Information Technology for Economic and Clinical Health (HITECH) Act. The Act authorized tens of billions of dollars in federal subsidies for hospitals and eligible physicians to adopt certified electronic health record (EHR) systems. To qualify for subsidy payments, eligible professionals and hospitals were required to certify that they met standards of the Meaningful Use program for EHR systems. Beginning in 2015, nonparticipation in Meaningful Use caused clinicians to incur a reduction in Medicare and Medicaid reimbursements.^1^

Meaningful Use originally comprised 3 stages, each of which required meeting increasingly comprehensive EHR adoption standards, as defined by the Centers for Medicare & Medicaid Services and the Office of the National Coordinator for Health IT, to qualify for incentive payments. Meaningful Use stage 1 set preliminary standards for the electronic recording and reporting of clinical information, requiring eligible clinicians to satisfy a set of 15 core objectives. Stage 2 increased the number of required EHR capabilities and encouraged clinicians to use this information to improve clinical processes. In so doing, Centers for Medicare & Medicaid Services and Office of the National Coordinator for Health IT sought to align the program with the National Quality Strategy.^2^ Stage 3 further increased these requirements and refined the Meaningful Use standards to focus on the improvement of health outcomes. The Meaningful Use program has since been replaced by the Promoting Interoperability program.^2^

The government’s role in incentivizing private investment and technological innovation has long been a subject of economic importance.^3,4,5^ Although the HITECH Act clearly accelerated hospitals’ adoption of EHRs,^6,7^ evidence regarding the Act’s association with other outcomes has been thin. Several researchers have found, at most, a small association with health or quality outcomes after more advanced adoption of health information technology (IT) capabilities, including EHR technology.^8,9,10^ Other researchers have documented meaningful reductions to costs for hospitals in IT-intensive areas beginning several years after EHR implementations^11^ and have found that hospital mortality statistics tend to improve after EHR implementations have had time to mature.^12^ Importantly, some have pointed to the unintended consequences of EHR adoption, such as an increased incidence of clinician burnout associated with the administrative load of EHR adherence and EHRs’ difficulty of use.^13,14,15^ Improving EHR usability might even improve professional satisfaction and patient health outcomes.^16,17^

To our knowledge, however, there has not been any empirical research on the association between the HITECH Act and innovation in related health care technologies. This is surprising, given that in the health care setting, major policy changes that lead to increased demand for health care products and services have been shown to increase innovative activities. Notable examples include studies of how pharmaceutical research and development and commercialization activities responded to demand increases created by the passage of Medicare Part D.^18,19^

In this economic evaluation study, we investigated whether the HITECH Act—which presented a large long-term increase in demand for and use of EHR systems—was associated with an increase in health care IT entrepreneurship in the years that followed. In particular, the large-scale digitization of the medical record system was not only a major project in itself, but it would have also created a large trove of digital health data and spurred new clinical workflows upon which other health care IT tools and products could be built. Given the overall ambivalent success of EHR implementations, including some apparent cost and quality benefits at the expense of widespread clinician dissatisfaction, burnout, and usability concerns, understanding how major public subsidies for health care IT have shaped investment compared with the broader entrepreneurial landscape will be valuable for guiding future investments in this area.

## Methods

We did not seek institutional review board approval for this evaluation because we did not use any individualized data, in accordance with 45 CFR §46. This study follows the Consolidated Health Economic Evaluation Reporting Standards (CHEERS) reporting guideline.^20^

To look for evidence of entrepreneurial finance flowing into the health IT sector, we examined the distribution of venture capital (VC) financing transactions, which typically are investments in young, privately held companies,^21^ for health care IT and EHR-related companies before and after the HITECH Act’s passage. We also compared investment patterns seen with those in the broader universe of VC transactions in the US. We interpreted seed-stage funding—that is, equity investments in companies in the earliest stages of financing—of private companies by VC firms as a proxy for entrepreneurship in the industry, whereby more seed-stage financing is an indicator of greater innovation in the space.

### Data

We collected data on VC investments in the US from Capital IQ, a comprehensive database that aggregates information on private investment transactions from public wire services and surveys of investment firms. Our data set includes the date, funding round, transaction value (when available), target company’s description, and target company's industry for each investment transaction.

Capital IQ includes a label for each transaction’s funding round. We categorized each fundraising round into one of 3 types: seed, early stage, and growth or late (see the eTable in the Supplement for detailed definitions of these categories). To determine whether companies’ products were associated with EHRs, we used the list of EHR search terms provided by the National Library of Medicine.^22^

### Statistical Analysis

We restricted our analysis to transactions between January 1, 2000, and August 31, 2018, for which the target company was incorporated and headquartered in the US. We also omitted the 31.3% of transactions for which the Capital IQ–recorded funding round was “venture,” “bridge,” or “debt.” We excluded bridge and debt transactions because they are typically not equity investments and do not fit clearly into the fundraising timeline of a typical VC-financed firm (bridge and debt, 9.5% of transactions). The “venture” label (21.8% of transactions), on the other hand, describes investments where the specific round is unknown. In robustness tests, we also included venture transactions in the group of non–seed-stage investments. After applying these criteria for inclusion, 70 982 total transactions remained. Of these, 1060 transactions involved companies with a primary industry classification of “health care industry software,” which we refer to as “health care IT” companies.

Finally, to determine whether companies’ products were related to EHRs, we matched the list of EHR keywords to the text of company descriptions included in the Capital IQ database. If any of the search terms were included in the company description, then we labeled the company as “EHR-related.” Of the 1060 health care IT transactions in our data set, we identified 333 as EHR-related.

To analyze whether the composition of the types of funding health care IT companies received changed in the period following the passage of the HITECH Act, we used a difference-in-differences design to test whether the fraction of investment transactions that raised seed-stage funding for health care IT companies increased more than that of control groups of companies in 3 categories: general health care (non-IT), IT (non–health care), and the entire universe of US VC transactions. The non-IT segment of health care companies and the non–health care segment of IT companies, in particular, are the most natural groups of investments to compare with health care IT investments, because they are likely to draw from the same set of expert investors, in particular those with a sector-specific investment mandate (eg, IT-focused VC firms or health care–focused VC firms). We coded the period from January 1, 2000, through February 17, 2009, as the pretreatment period, and February 18, 2009, through August 31, 2018, as the treatment period.

The outcome variable in our regressions was a binary variable indicating whether a given investment was seed stage, and the independent variables included whether the investment was in the post-HITECH period, whether the investment was in health care IT, an interaction term for these 2 variables, and a running time variable. We estimated this regression using a linear probability model for ease of interpretation.^23,24^ In robustness tests, we also implemented a logit specification. To calculate statistical significance, we used a 2-sided *t* test, with *P* < .05 denoting statistical significance.

All of our calculations were performed using R statistical software version 3.5.1 (R Project for Statistical Computing), and all figures were made using the ggplot2 library.^25,26^ Data were analyzed from September 2018 to August 2019.

## Results

Table 1 shows a breakdown of the number of VC, non–health care IT, health care IT, and EHR-related investments seen both before and after the passage of the HITECH Act. Our final analysis included 70 982 investments, of which 9425 (13.3%) were seed stage, 10 706 (15.1%) were early stage, and 50 851 (71.6%) were growth stage. Of the seed-stage investments (ie, those representing the earliest form of investment transaction and, thus, likely the youngest entrepreneurial firms), 1046 (11.1%) were in the pretreatment period and 8379 (88.9%) followed the HITECH Act in the treatment period.

**Table 1.** Transactions by Funding Round, Before and After the HITECH Act

Company Type, Funding Round Group	Transactions, No. (%)	
	Before HITECH	After HITECH
Electronic health record		
Seed	3 (2.5)	33 (15.5)
Early	24 (20.0)	29 (13.6)
Growth or late	93 (77.5)	151 (70.9)
Health care information technology		
Seed	10 (2.9)	170 (23.8)
Early	71 (20.6)	112 (15.7)
Growth or late	264 (76.5)	433 (60.6)
All venture capital (non–health care information technology)		
Seed	1036 (3.8)	8209 (19.2)
Early	4633 (17.1)	5890 (13.8)
Growth or late	21436 (79.1)	28718 (67.1)
All venture capital		
Seed	1046 (3.8)	8379 (19.2)
Early	4704 (17.1)	6002 (13.8)
Growth or late	21700 (79.1)	29151 (67.0)

Abbreviation: HITECH, Health Information Technology for Economic and Clinical Health.

**Figure 1.**
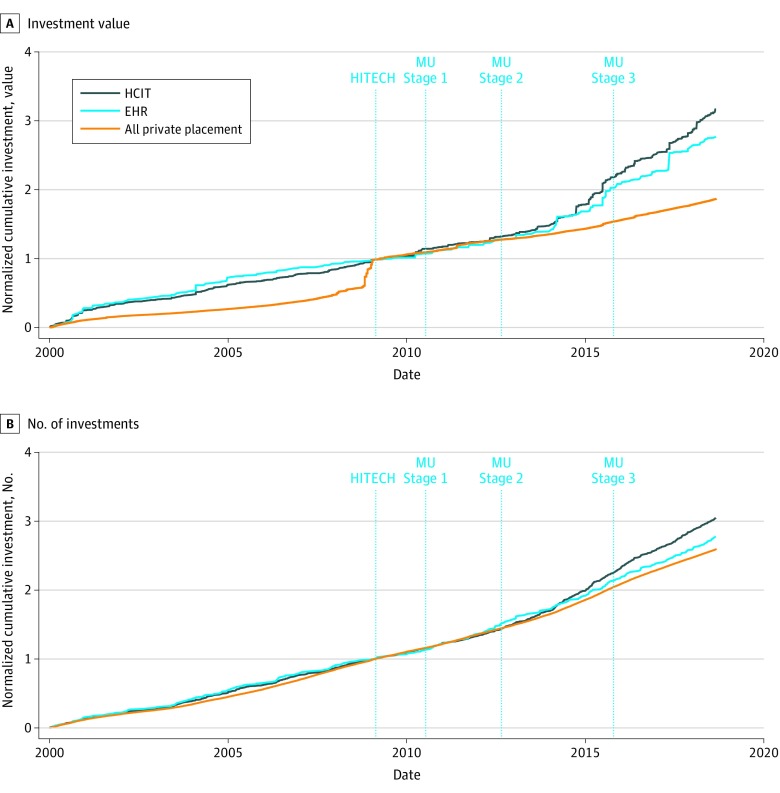
Investments in Health Care Information Technology (HCIT), Electronic Health Record (EHR) Technology, and All Other Private Placements Before and After Passage of the Health Information Technology for Economic and Clinical Health (HITECH) Act A and B, Graphs show the value (A) and number (B) of investments in HCIT, EHR technology, and all private investments beginning in 2000, normalized on the day the HITECH Act passed. MU indicates Meaningful Use.

Figure 1 shows cumulative investment in health care IT, EHR-related companies, and all VC beginning in 2000, normalized on the day the HITECH Act passed. Raw investment figures for health care IT and EHR-related investment are presented in eFigure 1 in the Supplement. Between January 1, 2000, and the passage of the HITECH Act, only $2.7 billion was invested in health care IT companies, compared with $6.2 billion between the Act and August 31, 2018. Furthermore, compared with the broader universe of VC investment, the number of investments in health care IT companies increased at a faster rate (12.4% vs 10.5% annualized), whereas the pace of investment in EHR-related companies stayed steady. Weighted by the dollar value of the transactions, however, investment in both health care IT companies and those we identify as EHR-related increased at a rate much faster (13.0% and 11.4%, respectively) than VC as a whole (6.9%) (Figure 1).

**Figure 2.**
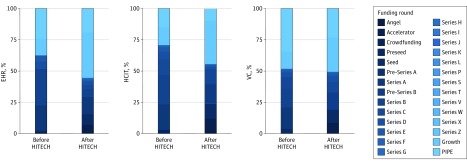
Funding Round Mix for Electronic Health Record (EHR)–Related, Health Care Information Technology (HCIT), and All Venture Capital (VC) Transactions Before and After Passage of the Health Information Technology for Economic and Clinical Health (HITECH) Act Lighter colors represent later-stage funding rounds, and darker colors represent earlier-stage funding rounds. In all 3 categories, the proportion of seed-stage investments (the darker segments of the chart) increased in the post-HITECH period. PIPE indicates private investment in public equity.

Figure 2 shows the funding round mix for EHR-related, health care IT, and all VC transactions in our sample before and after the HITECH Act. In all 3 categories, the proportion of seed-stage investments increased in the post-HITECH period. This shift, though, is most pronounced among the broader category of health care IT and is somewhat attenuated for the subset of companies we identify as EHR-related.

Table 2 extends this comparison, displaying the results of the fitted difference-in-differences regressions. After controlling for the positive linear time trend, health care IT companies saw an additional 5.1% (SE, 2.2%; 95% CI, 0.8% to 9.3%; *P* = .02) and 13.6% (SE, 1.9%; 95% CI, 9.9% to 17.2%; *P* < .001) probability of transactions being seed-stage compared with the entire sample of VC transactions and with non-IT health care VC transactions, respectively. In the comparison with non-health IT, however, health care IT had essentially 0 increased probability of a transaction being seed stage (−0.8% probability; SE, 2.4%; 95% CI, −5.4% to 3.9%; *P* = .75), suggesting that the trajectory of seed-stage investment in health care IT companies was similar to that of other non–health care IT investments, both before and after the HITECH Act.

**Table 2.** Fitted Difference-in-Differences Regression Coefficients of Seed-Stage Investment Probability by Industry, Compared With HCIT^a^

Variable	Regression coefficient, mean (SE)^b^		
	All venture capital	Health (non–information technology)	Information technology (nonhealth)
After HITECH	0.095 (0.005)^c^	0.030 (0.009)^c^	0.169 (0.01)^c^
HCIT	−0.007 (0.018)	−0.008 (0.015)	−0.015 (0.019)
After HITECH × HCIT^d^	0.051 (0.022)^e^	0.136 (0.019)^f^	−0.008 (0.024)^g^
*t*	0.006 (0.0005)^c^	0.004 (0.001)^c^	0.005 (0.001)^c^
Intercept	0.010 (0.003)^c^	0.018 (0.005)^c^	0.25 (0.005)^c^
Observations, No.	70 982	15 580	26 153
*R*^2^	0.052	0.03	0.083

Abbreviations: HCIT, health care information technology; HITECH, Health Information Technology for Economic and Clinical Health.

^a^ Each column compares the change in the proportion of seed-stage investments in HCIT companies with the change for a control group: the “all venture capital” column shows the comparison to the entire set of venture investments, and the “health (non–information technology)” and “information technology (nonhealth)” columns show the comparison with non–information technology health care investments and non-HCIT investments, respectively.

^b^ The dependent variable in all regressions is the probability of an investment being seed stage.

^c^ *P* < .01.

^d^ This row shows estimates for the primary association of interest, the additional fraction of health care IT seed-stage investments after the HITECH Act.

^e^ *P* = .02.

^f^ *P* < .001.

^g^ *P* = .75.

A crucial underlying assumption of the difference-in-differences design is that the outcome variable for the treatment and control groups would have evolved in parallel in the absence of the treatment. Although this assumption is not precisely testable, we plotted the fraction of investments that were seed stage for health care IT and the rest of the VC universe in 2-year intervals around the date the HITECH Act passed (eFigure 2 in the Supplement). Visually, the parallel trends assumption appears to hold: before the HITECH Act, the fraction of investments that were seed stage within health care IT and the rest of VC moved in tandem, only decoupling in the periods following the Act’s passage. Our results were also qualitatively robust to including VC transactions in the group of non–seed-stage investments and to the use of a logit regression specification.

## Discussion

In this study, we analyzed the association of a large federal incentive program for EHR adoption with subsequent indicators of entrepreneurship in health care IT. We found that, in the years after the program’s enactment, investments in health care IT and EHR-related companies increased at a rate much faster (13.0% and 11.4%, respectively) than VC as a whole (6.9%). In addition, the share of VC investments in seed-stage companies increased by 5.1% compared with trends in the broader VC investment landscape, suggesting that entrepreneurial activity in this sector became more attractive after the HITECH Act. To put the magnitudes of this association in perspective, for the near-decade leading up to the HITECH Act, fewer than 3% of health care IT transactions involved seed-stage financing. Furthermore, our results suggest that investment and entrepreneurship trends in the health care IT industry operated more similarly to those seen throughout the IT sector than those seen in health care investing more broadly. A decade after the HITECH Act, these results are important not only for understanding the full scope of the outcomes associated with the HITECH Act, but also for understanding how public policies may stimulate innovation writ large.

As seen in Figure 1, there was a notable increase in overall VC funding around the time of the HITECH Act. We hypothesize that this sharp, 1-time increase in the pace of private placements is associated with the contemporaneous economic recession and recovery as investors reached for yield through riskier investment strategies. However, a formal analysis is beyond the scope of this article.

Our analysis does not imply that the HITECH Act or its subsequent implementation per se caused a shift in the type of health care IT companies that receive VC financing, nor can we claim a particular mechanism by which this may have occurred; several explanations are plausible. For example, a shift in unobserved investor demand for seed-stage health care IT investments may have been coincident with, or an input into, the Obama administration’s desire to upgrade nationwide health care infrastructure; ostensibly unrelated, contemporaneous changes in the IT landscape (eg, the advent of cloud computing) may have induced greater opportunity for innovation in IT broadly (including health care IT); or, by accelerating the adoption of EHR systems, the HITECH Act may have, as a second-order consequence, created the opportunity for would-be entrepreneurs in the health care IT industry to build business models that rely on EHRs for data, processes, and/or customers.

Entrepreneurship, marked by the formation and funding of early-stage companies, has long been recognized as an important input into or proxy for innovation.^27,28,29^ By spurring adoption of EHRs and digitization of clinician workflows (ie, automated collection of clinical data, clinical decision support capabilities, computerized order entry, and other software-driven tools) the HITECH Act represented a substantial opportunity for demand-pull innovation in health care IT, in which greater market-wide demand for EHR capabilities in the US spurred research and innovation in the industry. The latter explanation of the experience of the post-HITECH VC industry suggests that the HITECH Act may have had a considerable association with the development of new technologies and, as a result, productivity in the health care industry. As EHR systems become more pervasive and functional, stimulated by the HITECH Act, new products can be built on top of those capabilities. Moreover, our results imply an important role for government incentive programs in promoting entrepreneurship around follow-on technologies in general, dovetailing with prior literature on the government’s capacity to use grants and other financial incentives to promote innovation.^30^

Critically, our results exist in the larger context of EHR implementation and usage realities in the US. Electronic health records have numerous benefits: they have been shown to have a positive association with certain costs, outcomes, and some measures of quality.^10,11,12,31,32^ Furthermore, EHRs enabled tremendous clinical research opportunities^33^ and fostered better population and public health management.^34^ Yet EHRs have also been problematic and are associated with increased costs,^35^ poor usability,^36^ intense clinician dissatisfaction,^15^ and other unintended consequences.^37^ Our results—specifically, that VC investments in EHR-related and health IT companies increased after the HITECH Act—may be disappointing vis-a-vis many of the challenges associated with contemporary EHRs, suggesting a misalignment of funding and needs. That the funding was skewed toward early-stage companies also suggests a nascent industry that may still take time to mature.

In 2018, the Meaningful Use program was renamed Promoting Interoperability, and the focus of the incentive program shifted toward interoperability and improving patient access to health data. Interoperability has been slow to advance as EHR adoption has accelerated,^38^ and as a result, interoperability has become a focal point of US policy efforts, along with improving patient access to health data.^39^ It is unclear how this shift in focus will be associated with entrepreneurship and overall HCIT investment, although we might expect the nature of the technology to evolve to reflect these priorities.

### Limitations

This study has several limitations. First, our study focused on VC investment; we are unable to address other funding streams that existing companies could have used to invest in EHR or other technologies without such external financing, such as a publicly traded company or a privately held company that invested in research and development. It is possible, for instance, that although our results do not show a marked increase in outside investment in VC-financed companies developing EHRs, existing companies have been able to finance these projects internally. In addition, we were unable to assess the outcomes for the companies that received funding (eg, how many hospital customers they had or how many health care clinicians used their products).

## Conclusions

This study suggests that the HITECH Act and its EHR adoption incentive program were associated with increased health care IT and EHR-related funding as well as increased early-stage entrepreneurship in the health care IT sector. These findings suggest that, although incentive programs in health care IT may serve as an important catalyst for entrepreneurship and innovative activity and should inform future policy efforts in this space, more work is needed to understand how the money from these incentive programs has been deployed and who has benefited from it.

## Notes

Supplement. **eTable.** Funding Round Categorization **eFigure 1.** Investment in EHR-Related and HCIT Companies **eFigure 2. **Parallel Trends Analysis
