# Supplementary material for: Association of the Meaningful Use Electronic Health Record Incentive Program With Health Information Technology Venture Capital Funding
Source: JAMA Netw Open. 2020 Mar 24;3(3):e201402. doi: 10.1001/jamanetworkopen.2020.1402 (PMC7093764; doi:10.1001/jamanetworkopen.2020.1402)
Supplement: Supplement. — eTable. Funding Round Categorization eFigure 1. Investment in EHR-Related and HCIT Companies eFigure 2. Parallel Trends Analysis [file jamanetwopen-3-e201402-s001.pdf]

Supplementary Online Content

Lite S, Gordon WJ, Stern AD. Association of the meaningful use electronic health record incentive program with health information technology venture capital funding. *JAMA Netw Open*. 2020;3(3):e201402. doi:10.1001/jamanetworkopen.2020.1402

eTable. Funding Round Categorization
eFigure 1. Investment in EHR-Related and HCIT Companies
eFigure 2. Parallel Trends Analysis

This supplementary material has been provided by the authors to give readers additional information about their work.

© 2020 Lite S et al. *JAMA Network Open.*

eTable. Funding Round Categorization

Group	Round Type (from Capital IQ)
Seed	Angel, Accelerator, Crowd-Funding, Pre-Seed, Seed
Early	Pre-Series A, Series A
Growth / Late	Pre-Series B, Series B, Series C, Series D, …, PIPE
Excluded	Venture, Debt, Bridge

© 2020 Lite S et al. *JAMA Network Open.*

eFigure 1: Investment in EHR-Related and HCIT Companies

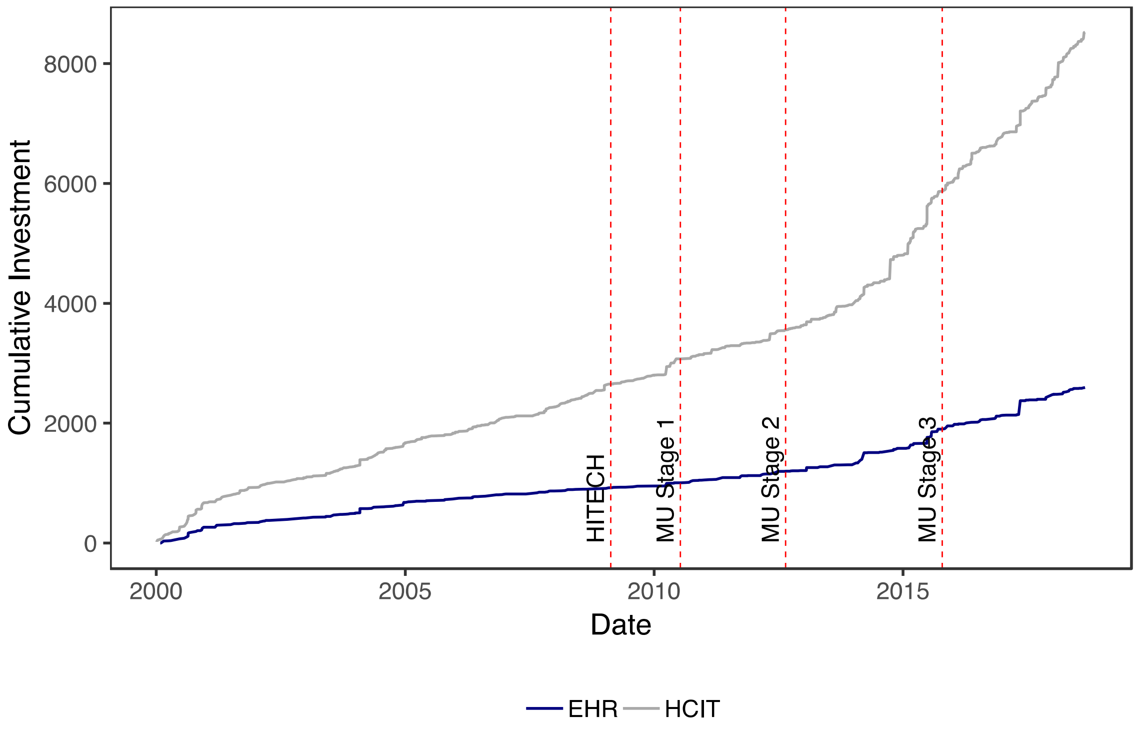

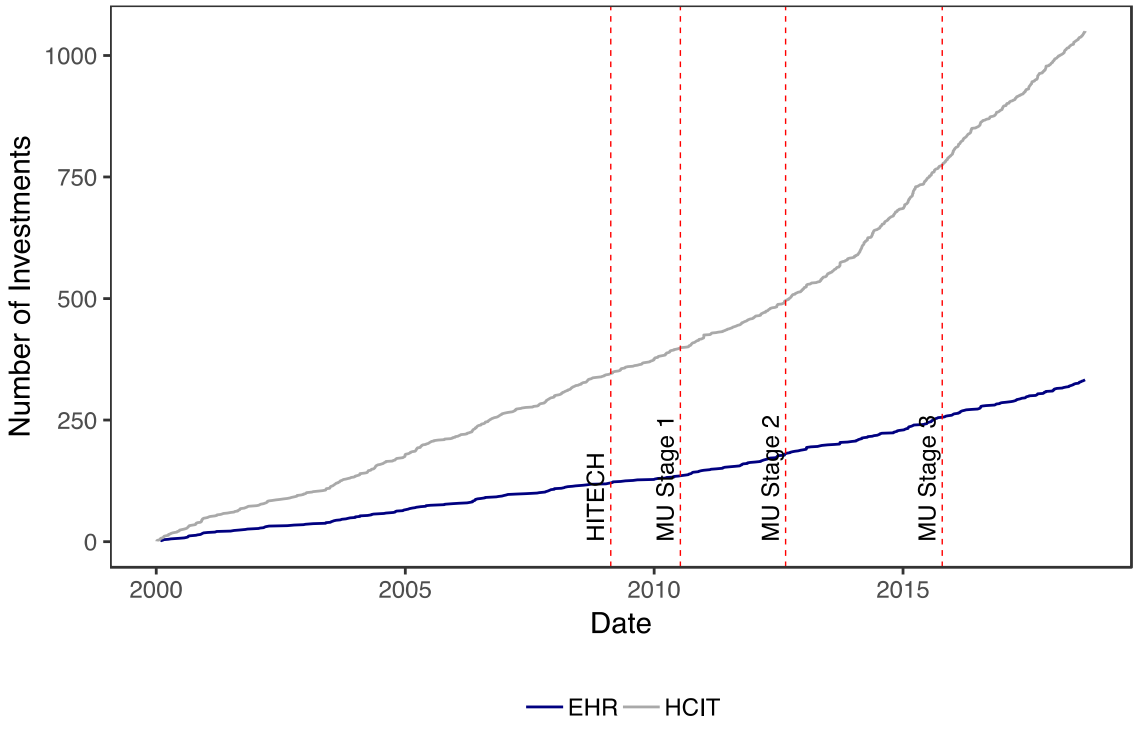

© 2020 Lite S et al. *JAMA Network Open.*

eFigure 2: Parallel Trends Analysis

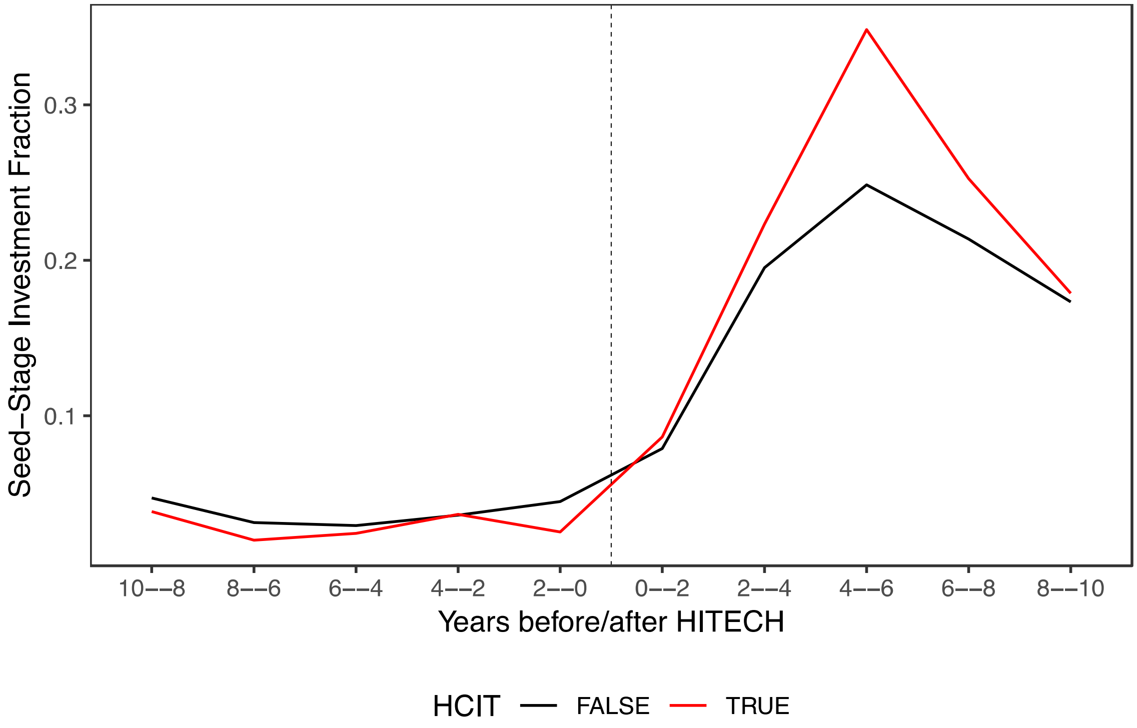

© 2020 Lite S et al. *JAMA Network Open.*
